# Detection of tumor-derived DNA dispersed in the airway improves the diagnostic accuracy of bronchoscopy for lung cancer

**DOI:** 10.18632/oncotarget.18159

**Published:** 2017-05-24

**Authors:** Taichiro Goto, Yosuke Hirotsu, Takahiro Nakagomi, Daichi Shikata, Yujiro Yokoyama, Kenji Amemiya, Toshiharu Tsutsui, Yumiko Kakizaki, Toshio Oyama, Hitoshi Mochizuki, Yoshihiro Miyashita, Masao Omata

**Affiliations:** ^1^ Lung Cancer and Respiratory Disease Center, Yamanashi Prefectural Central Hospital, Yamanashi, Japan; ^2^ Genome Analysis Center, Yamanashi Central Hospital, Yamanashi, Japan; ^3^ Department of Pathology, Yamanashi Central Hospital, Yamanashi, Japan; ^4^ University of Tokyo, Tokyo, Japan

**Keywords:** bronchial wash, bronchoscopy, lung cancer, mutation, next-generation sequencing

## Abstract

The diagnostic accuracy of bronchoscopy for detecting lung cancer, especially peripheral lung cancer with lesions outside the endoscopically visible range, remains unsatisfactory. The aim of this study was to perform next-generation sequencing on bronchoscopic specimens to determine whether this improves the accuracy of bronchoscopy for diagnosing lung cancer and to identify factors influencing sensitivity. The bronchoscopic sensitivity for diagnosing lung cancer was initially evaluated in 191 patients who underwent lobectomy after bronchoscopy at our hospital. Sputum, bronchial wash fluid, and resected lung cancer specimens were subsequently collected from 18 patients with peripheral small cell lung cancer for genomic analysis. DNA was extracted from formalin-fixed, paraffin-embedded surgical tissue specimens and the supernatant and cell fractions of sputum and bronchial wash fluid. Deep sequencing was performed using a lung cancer panel covering all exons of 53 lung cancer-related genes. The bronchoscopic sensitivity for diagnosing lung cancer at our hospital was 60.7%. Multivariate analysis revealed that this was influenced by tumor size and location, but not histological type or lymph node metastasis. The sensitivity was the highest for biopsy followed by curettage and bronchial wash specimens. DNA mutations homologous to those identified in the primary lesions were detected in the bronchial wash fluid of 10 patients (55.6%), while only 2 patients (11.1%) were diagnosed with lung cancer based on conventional cytological examinations. In conclusion, the addition of genomic analysis to routine pathological examinations improves the diagnostic accuracy of bronchoscopy.

## INTRODUCTION

Recently, the incidence of central-type squamous cell lung cancer has been decreasing worldwide, whilst that of peripheral lung adenocarcinoma has been increasing. Consequently, a great proportion of lung cancer lesions are outside the endoscopically visible range, making a diagnosis technically challenging [1, 2]. Bronchoscopic examination is an uncomfortable procedure that has limited sensitivity (range, 34.0−88.0%) [2]. During bronchoscopy, curettage or biopsy forceps are brought in close proximity to the tumor under fluoroscopic guidance. However, there is no means of secure reaching to the tumor.

We retrospectively analyzed the patient medical records at our hospital to assess the sensitivity of bronchoscopy for diagnosing lung cancer and to identify factors influencing sensitivity. During the bronchoscopy procedure, curettage, biopsy, and washing are performed, in this order, to make a complete diagnosis. In this study, accurate diagnostic performance was compared between the three specimen types to try to elucidate areas for improvement in bronchoscopic examinations.

Cell-free circulating tumor DNA (ctDNA) in plasma or other body fluids can potentially serve as a non-invasive surrogate biomarker in cancer diagnosis and treatment [3-7]. CtDNA comprises small nucleic acid fragments that are released from tumor cells into the blood through a variety of cellular events (e.g., apoptosis, necrosis, and secretion) [5]. CtDNA is detectable in the plasma and serum of advanced-stage cancer patients [3, 8-12]. Therefore, so-called “liquid biopsy” is one of the most immediate applications of ctDNA [4, 5, 9, 13]. Lung cancer is typically diagnosed by histopathological or cytological examinations of bronchoscopic specimens. Detection of dispersed DNA in the airway by next-generation sequencing can potentially offer a more accurate method for diagnosing lung cancer.

In this study, we hypothesize that cell-free ctDNA with mutant alleles is released from cancer cells and dispersed into the airway. We evaluated the diagnostic accuracy of bronchoscopic liquid biopsy using dispersed DNA for detecting lung cancer.

## RESULTS

### Patient characteristics

Of the 191 patients enrolled in this study, 115 (60.2%) were men and 76 (39.8%) were women (mean age, 70.5 ± 7.8 years). One hundred and fifty-one patients had adenocarcinoma, 32 patients had squamous cell carcinoma (SCC), and 8 patients had other types of lung malignancies (Table 1). The mean size of the tumors was 31.5 ± 5.6 mm.

**Table 1:** Patient characteristics

Characteristic	Patients (*n* = 191)
Age (years), mean (range)	70.5 (44-90)
Sex, *n* (%)	
M	115 (60.2)
F	76 (39.8)
Tumor size (cm), *n* (%)	
<2.0	41 (21.4)
2.0-3.0	71 (37.2)
>3.0	79 (41.4)
Histology, *n* (%)	
ADC	151 (79.1)
SCC	32 (16.7)
Other	8 (4.2)
Tumor location, *n* (%)	
Central	20 (10.5)
Middle	42 (22.0)
Peripheral	129 (67.5)
pStage, *n* (%)	
I	115 (60.2)
II	19 (10.0)
III	52 (27.2)
IV	5 (2.6)

ADC, adenocarcinoma; M, male; F, female; pStage, pathological stage.

### Retrospective analysis of bronchoscopic sensitivity for the diagnosis of lung cancer

Conventional bronchoscopy detected lung cancer in 116 of the 191 patients, with a diagnostic sensitivity of 60.7%. Multivariate analyses identified tumor size and location as factors influencing diagnosis (*P* = 0.002 and *P* = 0.03, respectively). In contrast, no associations were observed for age (*P* = 0.71), sex (*P* = 0.82), histological type (*P* = 0.41), pathological stage (*P* = 0.14), or the occurrence of lymph node metastases (*P* = 0.13). A comparison of the clinical characteristics between patients with and without a correct diagnosis of lung cancer revealed significant differences in tumor size and location. No significant differences were observed for any other factors (Supplementary Table S1). The diagnostic sensitivities for tumors measuring < 2.0 cm, 2.0-3.0 cm, and > 3.0 cm in diameter were 29.3%, 54.9%, and 81.0%, respectively (Table 2). Therefore, sensitivity increased in relation to tumor diameter. The diagnostic sensitivities for central, middle, and peripheral lung cancers were 80.0%, 73.8%, and 53.4%, respectively (Table 2). Thus, sensitivity was greater for more centrally located tumors.

**Table 2:** Bronchoscopy sensitivity

Characteristic	Eligible patients (*n*)	Patients with correct Dx (*n*)	BF sensitivity (%)
All patients	191	116	60.7
Tumor size (cm)			
<2.0	41	12	29.3
2.0-3.0	71	39	54.9
>3.0	79	64	81.0
Tumor location			
Central	20	16	80.0
Middle	42	31	73.8
Peripheral	129	69	53.4

Dx, diagnosis; BF, bronchofiberscopy.

Multivariate analyses revealed odds ratios that were 2.91-fold (95.0% confidence interval [CI]: 1.31-7.60) and 9.68-fold (95.0% CI: 3.90-25.55) higher for the detection of tumors measuring 2.0-3.0 cm or > 3.0 cm in diameter relative to the detection of tumors measuring < 2.0 cm, which was used as a reference (Table 3). When peripheral lung cancer was used as a reference for tumor location, the odds ratio for the diagnostic sensitivity for central lung cancer was 5.06-fold higher (95.0% CI: 1.43−13.68; Table 3).

**Table 3:** Odds ratios of correct diagnostic performance of bronchoscopy

Characteristic	OR (95.0% CI)	*P*-value
Tumor size (cm)		
<2.0	1 (Ref.)	
2.0−3.0	2.91 (1.31−7.60)	0.017^*^
>3.0	9.68 (3.90−25.55)	<0.001^*^
Tumor location		
Peripheral	1 (Ref.)	
Middle	1.77 (0.90−4.96)	0.160
Central	5.06 (1.43−13.68)	0.014^*^

*P < 0.05

OR, odds ratio; CI, confidence interval; Ref., reference

### Comparison of the diagnostic sensitivity for adenocarcinoma *versus* SCC

Univariate analyses revealed that the diagnostic sensitivity for SCC was significantly higher than that for adenocarcinoma (82.6% *vs.* 53.1%; *P* = 0.04, Chi-square test). The mean size of the tumors was 39.5 ± 6.8 mm for SCC and 28.7 ± 5.6 mm for adenocarcinoma (*P* = 0.007). Moreover, the proportion of tumors located in the central lung field was significantly higher in SCC patients than in adenocarcinoma patients (*P* = 0.04, Chi-square test).

### Differences in diagnostic sensitivities between different specimen types

**Figure 1:**
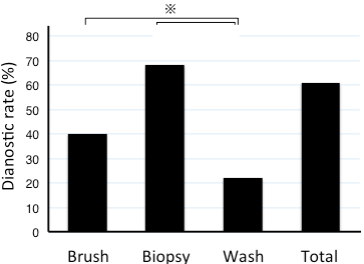
Diagnostic sensitivity of bronchoscopic specimens The diagnostic yield was lower for bronchial wash than curettage or biopsy specimens. The asterisk denotes a *P* < 0.05.

The diagnostic yields for lung cancer according to bronchoscopic method were 40.2%, 68.8%, and 21.8% for curettage, biopsy, and bronchial wash fluid, respectively (Figure 1). Thus, the diagnostic yield was lower for bronchial wash fluid than other specimen types (*P* < 0.05, Chi-square test). Sixty-five percent of patients diagnosed with lung cancer based on curettage or biopsy specimens were negative by examination of bronchial wash specimens. Conversely, only 5.0% of patients who were negative by examination of curettage or biopsy specimens were diagnosed as having lung cancer by examination of bronchial wash specimens.

### Case presentations

**Figure 2:**
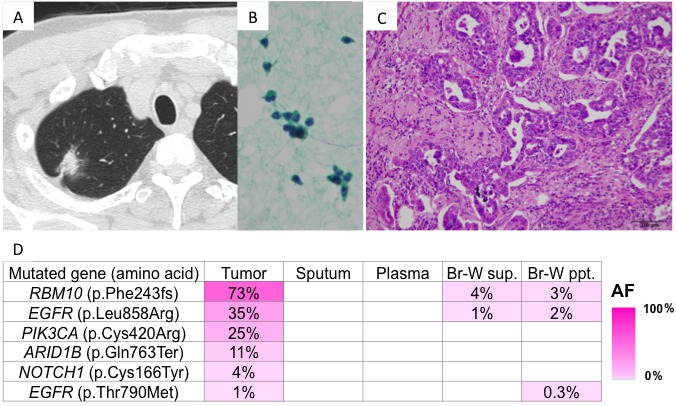
Presentation of case 1 **A**. CT findings: A nodule with an irregular surface and pleural indentation was present in right segment 1; **B.** Cells showed no atypia on Papanicolaou staining; and **C.** Histologically, an acinar pattern of adenocarcinoma was observed on hematoxylin and eosin staining. **D.** Genomic analyses: Heat map of the mutations detected in each sample. The left column lists the mutated genes with the corresponding amino acid changes. Some mutations were detected in both the primary tumor and bronchial wash specimens. AF, allele fraction; Br-W, bronchial wash; sup., supernatant; ppt., precipitant.

Case 1 was a 64-year-old man who was a smoker. Chest X-ray screening revealed a tumor shadow in the upper field of the right lung. A computed tomography (CT) scan revealed a tumor in right segment 1 (Figure 2A). Bronchoscopy and endobronchial ultrasound were performed. Although examination of curettage and bronchial wash specimens indicated cytological class I (Figure 2B), adenocarcinoma was diagnosed by punch biopsy. A right upper lobectomy was performed. The tumor was pathologically diagnosed as a Stage IB invasive adenocarcinoma (Figure 2C). Genomic analysis of the bronchial wash fluid identified mutations in the RNA-binding motif protein 10 (p.Phe243fs) and epidermal growth factor receptor (*EGFR*) (p.Leu858Arg and p.Thr790Met) genes in the primary lesion. Mutations were detected in both the supernatant and cell fraction of the bronchial wash specimen (Figure 2D), but not in the sputum or plasma. *EGFR* p.Thr790Met is a resistance mutation that is well-known for emerging after the administration of EGFR-tyrosine kinase inhibitors. However, since EGFR-tyrosine kinase inhibitors were not administered in this case, the mutation was considered a *de novo* p.Thr790Met.

**Figure 3:**
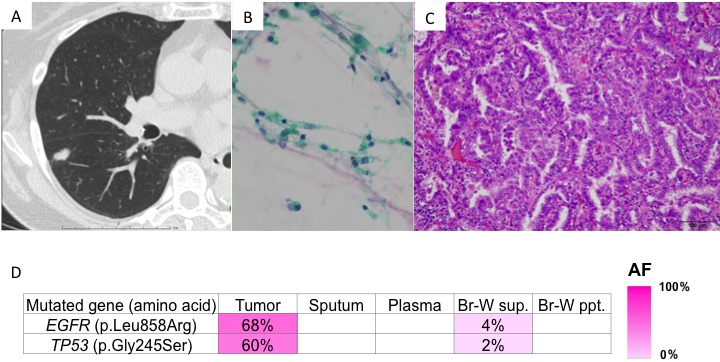
Presentation of case 2 **A**. CT findings: A nodule was located in right segment 6; **B.** Cells showed no atypia on Papanicolaou staining; and **C.** Histologically, a lepidic pattern of adenocarcinoma was observed on hematoxylin and eosin staining. **D.** Genomic analyses: Heat map of the mutations detected in each sample. The left column lists the mutated genes with the corresponding amino acid changes. The mutations were detected in both the primary tumor and bronchial wash supernatant. AF, allele fraction; Br-W, bronchial wash; sup., supernatant; ppt., precipitant.

Case 2 was a 50-year-old man who was a former smoker. CT screening revealed a tumor in right segment 6 (Figure 3A). Bronchoscopy and endobronchial ultrasound were performed. Although examination of the bronchial wash specimen indicated cytological class I (Figure 3B), adenocarcinoma was diagnosed by examination of curettage and punch biopsy specimens. A right lower lobectomy was performed. The tumor was pathologically diagnosed as a Stage IA invasive adenocarcinoma (Figure 3C). Genomic analysis of the bronchial wash fluid identified mutations in the *EGFR* (p.Leu858Arg) and tumor protein 53 (p.Gly245Ser) genes in the primary lesion. Mutations were detected in the supernatant of the bronchial wash specimen (Figure 3D), but not in the cell fraction of the bronchial wash specimen, sputum, or plasma.

### Diagnosis of lung cancer by examination of bronchoscopic specimens: a comparison between microscopic pathology and genomic analysis

Of the 18 patients in the liquid biopsy study, 14 (77.8%) were men and 4 (22.2%) were women (mean age, 70.1 ± 8.1 years). Fifteen patients (83.3%) were smokers and 3 patients (16.7%) were non-smokers. Fourteen patients had adenocarcinoma, 3 patients had SCC, and 1 patient had small cell carcinoma. The mean size of the tumors was 21.7 ± 6.9 mm (Supplementary Table S2).

Based on conventional cytological analysis of bronchial wash specimens, 2 patients (11.1%) were diagnosed with lung cancer. One patient had class IIIB and the other patient had class V. The remaining 16 patients had non-diagnostic cytological specimens, including 9 patients with class I, 5 patients with class II, and 2 patients with class III. Genomic analysis of the supernatant and precipitate of the bronchial wash specimens revealed DNA mutations homologous to those in the primary lesion in 10 patients (55.6%) (Table 4 and Supplementary Table S3). The diagnostic yield of the genomic analysis was superior to that of the pathological examinations based solely on the bronchial wash specimens and not the curettage or biopsy specimens (Table 4). Examinations of sputum and plasma specimens led to a diagnosis of lung cancer in 1 patient, with a diagnostic yield of 5.6%. In this patient, mutant DNA homologous to that of the primary lesion was detected in sputum collected before bronchoscopy (Case 3 in Table 4 and Supplementary Table S3). Although findings from the routine pathological examinations were inconclusive, the patient was diagnosed with lung cancer by genomic analysis.

**Table 4:** Conventional and genomic bronchoscopic diagnoses of lung cancer

Case	1	2	3	4	5	6	7	8	9
Microscopic Dx									
Curettage	−	+	−	+	−	+	−	+	+
Punch bx	+	+	−	+	−	+	N/P	+	−
Wash	−	−	−	−	−	−	−	+	−
Genomic Dx									
Wash sup.	+	+	+	−	−	−	−	+	+
Wash ppt.	+	−	−	−	−	−	−	−	+
Sputum	−	−	+	−	−	−	−	−	−
Plasma	−	−	+	−	−	−	−	−	−
**Case**	**10**	**11**	**12**	**13**	**14**	**15**	**16**	**17**	**18**
Microscopic Dx									
Curettage	−	−	−	+	+	−	−	+	−
Punch bx	+	−	+	+	N/P	−	−	−	−
Wash	−	−	−	−	−	−	−	+	−
Genomic Dx									
Wash sup.	+	+	−	+	+	−	−	+	−
Wash ppt.	+	+	−	−	+	−	−	−	−
Sputum	−	−	−	−	−	−	−	−	−
Plasma	−	−	−	−	−	−	−	−	−

+: Cancer was diagnosed. −: Cancer was not diagnosed.

For cytological analysis, the minus sign (−) indicates classes I, II, and III, whereas the plus sign (+) indicates classes IIIb, IV and V. Cases 5 and 7 were histologically diagnosed as adenocarcinoma in situ. Dx, diagnosis; bx, biopsy; sup., supernatant; ppt., precipitant; N/P, not performed

Overall, routine pathological examinations yielded a diagnosis of lung cancer in 11 patients (61.1%). By including genomic analysis, the diagnostic yield was increased to 13 patients (72.2%). In the 5 undiagnosed patients, the mean size of the tumors was as small as 14.6 ± 6.9 mm. Furthermore, 2 patients exhibited pure ground-glass opacity on imaging findings and were confirmed as having adenocarcinoma *in situ* based on pathological examinations (Table 4 and Supplementary Table S2).

## DISCUSSION

In this study, we demonstrate that tumor location and size are important factors for the bronchoscopic diagnosis of lung cancer. Although the current bronchoscopic diagnostic yield for lung cancer is unsatisfactory, it may be improved by including genetic analyses of bronchoscopic specimens. We also demonstrate that, in addition to ctDNA in plasma, DNA released from cancer cells is dispersed in the airway.

The findings of our retrospective analysis reveal the importance of tumor size and location, which is consistent with previous reports [1, 2, 14]. Although the bronchoscopic diagnostic yield differed between adenocarcinoma and SCC patients, this may be attributable to the fact that there is a high probability of tumor size and location being confounding factors. Thus, histological type and vessel invasion are not the most important factors for tumor diagnosis. Instead, being able to reach the tumors with forceps is critical. Various techniques and tools have been developed for aiding the passage of forceps through multiple divergent bronchi to reach the tumor, including virtual navigation, an ultra-thin fiberscope, and endobronchial ultrasound. The full application of these approaches in our institution has led to a gradual improvement in the accuracy of lung cancer diagnoses (data not shown).

Nevertheless, conventional diagnostic methods have failed to establish a preoperative diagnosis in approximately 40.0% of patients at our hospital who underwent surgery for suspected lung cancer. We speculated that the diagnostic accuracy of lung cancer could be improved by including high-level genetic analyses of the obtained specimens, rather than developing more reliable techniques for reaching the tumors with forceps.

In previous studies [1, 15], the analysis of messenger RNA expression in mainstem bronchi with normal appearances revealed that the gene expression classifier had improved the diagnostic performance of bronchoscopy for the detection of lung cancer. In the present study, instead of examining messenger RNA expression in normal cells surrounding the tumors, we attempted to detect DNA released by tumor cells to determine whether this approach had the potential to offer greater diagnostic specificity.

In the present study, we reveal that among the conventional pathological diagnostic measures, the contribution of bronchial wash fluid to the diagnosis of lung cancer was extremely limited. In 65.0% of patients who were diagnosed with lung cancer by examination of curettage or biopsy specimens, no cancer cells were detected in the bronchial wash fluid. This suggests that the significance of the cytological findings in the bronchial wash fluid is currently limited. However, given that lung cancer arises from the epithelium, we hypothesized that compared to plasma ctDNA, DNA dispersed into the airway would be more abundant, and therefore more readily detected. We speculated that cancer-specific mutations detected in the bronchial wash fluid could potentially be useful for the diagnosis of lung cancer.

DNA mutations homologous to those identified in primary lesions were detected in the supernatant or precipitate of the bronchial wash fluid of 10 and 5 patients, respectively. In other words, in 5 patients, mutant DNA was detected only in the supernatant and not in the cell fraction of the bronchial wash fluid. This suggests that mutant DNA dispersed in the epithelial lining fluid of the airway is more important for lung cancer diagnosis than those in cancer cells which is released into the airway. Additionally, DNA mutations homologous to those identified in primary lesions were detected in the supernatant of the bronchial wash fluid and peripheral plasma of 10 and 1 patients, respectively. In other words, in 9 patients, mutant DNA was detected only in the supernatant of the bronchial wash fluid and not in the peripheral blood. These findings suggest that mutant DNA dispersed in the airway is more important for lung cancer diagnosis than that which is detected in peripheral blood.

Detecting mutant DNA in the supernatant of the bronchial wash fluid may represent a new technique for the bronchoscopic diagnosis of lung cancer. When only bronchial wash specimens were examined, lung cancer was diagnosed through routine pathological examinations in 2 (11.1%) of 18 patients, whereas 10 (55.6%) of the 18 patients were diagnosed by genomic analysis. A significant difference in diagnostic yield was observed. Moreover, in conventional cytological examinations, specimens were subjectively assigned by pathologists to one of five cytological classes (I-V), which can yield variable results. In contrast, genomic analysis relies on objectively collected data and the results are more precise. Herein, we demonstrate that the bronchial wash fluid, whose diagnostic contribution has been regarded as limited, can be effectively utilized for precision diagnosis by the recent advent of “deep sequencing” methods.

In the present study, the clinical application of genomic analysis was only assessed for peripheral small cell lung cancer. We believe that this method will be more broadly applicable when applied to lung cancers arising from more central locations. The fact that there was also a patient (Case 3) in whom mutant DNA was detected in the sputum collected before bronchoscopy suggests that mutant DNA is continually being released from some types of primary lesions, even in the absence of mechanical treatments (e.g., bronchoscopy). Thus, genomic analysis may represent an appropriate non-invasive tool for the diagnosis of lung cancer.

This study has several limitations. First, the physiology and dispersion rate of mutant DNA into the airway has yet to be elucidated, and second, the sample sizes were relatively small. Therefore, further studies are warranted to confirm whether the detection of dispersed mutant DNA can be reliably and broadly used for the diagnosis of lung cancer. Nevertheless, our findings clearly indicate that next-generation sequencing is an additional diagnostic tool for so-called “precision medicine”. We anticipate that the dispersion of mutant DNA into the airway may be clinically applicable not only to the diagnosis of lung cancer, but also for monitoring tumor dynamics over time, the detection of residual disease, and the emergence of resistance mutations.

## MATERIALS AND METHODS

### Study population

The medical records of 191 consecutive patients with primary lung cancer who underwent preoperative bronchoscopic evaluation and surgical resection at our hospital between January 2010 and October 2015 were retrospectively reviewed. The patients’ data were obtained from the cancer registry database of our institution. Information collected from the patients’ medical records included preoperative characteristics, CT findings (tumor size and location), the operative procedure, and histopathological diagnosis, including the presence or absence of lymph node metastases.

Peripheral, central, and middle lung cancers were defined as cancers with primary lesions located in the outer, inner, or middle one-third of the lung field, respectively. Tumor size was measured as the maximum diameter on CT images acquired prior to bronchoscopy. Histological typing was performed according to the World Health Organization’s classification (third edition) [16] and clinical staging was performed according to the International Union Against Cancer tumor-node-metastasis classification (seventh edition) [17].

### Conventional bronchoscopic evaluation

During bronchoscopy, routine curettage, biopsy, and washing were performed, in this order, using a thin bronchoscope. Some patients did not undergo biopsy, owing to the occurrence of bleeding and cough reflex during the bronchoscopy procedure. In instances where the biopsy forceps failed to reach the tumor, only curettage and washing were performed. The specimens were diagnosed by two specialist pulmonary pathologists.

### Pathological evaluation of the resected specimens

One hundred and ninety-one patients underwent lobectomy for diagnosed or suspected lung cancer following bronchoscopy. All of the resected specimens were histologically confirmed as lung cancer from postoperative pathological examinations. In a retrospective analysis, the diagnostic sensitivity of preoperative bronchoscopy was evaluated in patients with a confirmed diagnosis of lung cancer. To identify factors affecting diagnostic sensitivity, the patients’ age and sex, tumor size and location, histological type, pathological stage, and presence or absence of lymph node metastases (determined by postoperative pathological findings) were evaluated in a multivariate analysis.

### Sample preparations for genomic analysis

In the liquid biopsy study, we selected the first 18 patients with peripheral lung tumors with a maximum diameter of ≤3.0 cm who underwent bronchoscopy followed by surgery between June and August 2015. All participants provided informed written consent prior to participation in the genetic research studies. Research was conducted in accordance with the Declaration of Helsinki and the study was approved by the Institutional Review Board Committee of Yamanashi Central Hospital (Yamanashi, Japan).

Pharyngeal anesthesia was administered immediately prior to bronchoscopy. The sputum coughed up at the time of anesthesia was used for mutation analysis. After thorough mixing of the bronchial wash specimens, small aliquots were used for genomic analysis and the rest were used for routine pathological examinations. For peripheral blood specimens, plasma ctDNA was probed and the lymphocyte genome was used as a normal control.

### Genomic analysis of the sample preparations

Bronchial wash was collected into sterile Falcon tubes (Corning Life Sciences, Tewksbury, MA, USA) and blood specimens were collected into EDTA-2Na tubes. Cell pellets and the buffy coat were isolated by centrifugation at 820 *g* and 25.0°C for 10 minutes. The supernatants from the bronchial wash and blood plasma were centrifuged at 20,000 *g* for 10 minutes before being transferred to sterile tubes and stored at −80.0°C until DNA extraction. Total DNA was extracted from lymphocytes and cell pellets using the QIAamp DNA Blood Mini Kit (Qiagen GmbH, Hilden, Germany). DNA was purified from blood plasma and the supernatant using the QIAamp Circulating Nucleic Acid Kit (Qiagen GmbH). DNA was also extracted from sputum using the QIAamp DNA Mini Kit (Qiagen GmbH), according to the manufacturer’s instructions. Serial sections of formalin-fixed, paraffin-embedded (FFPE) tissue were stained with hematoxylin and eosin. The sections were microdissected using an ArcturusXT laser-capture microdissection system (Thermo Fisher Scientific Inc., Waltham, MA, USA) and tumor DNA was extracted using the QIAamp DNA FFPE Tissue Kit (Qiagen GmbH).

### Quantitative real-time PCR assessment of DNA integrity

DNA fragmentation in FFPE DNA samples was assessed using the TaqMan RNase P Detection Reagents Kit and FFPE DNA QC Assay on a ViiA7 Real-Time PCR instrument (Thermo Fisher Scientific Inc.). Human control genomic DNA (included in the TaqMan RNase P Detection Reagents Kit) was serially diluted 4.0-fold to generate a five-point standard curve that was used to determine absolute DNA concentrations. DNA fragmentation was estimated as the ratio of DNA (relative quantification) obtained for the long (256 bp) *versus* short (87 bp) amplicon, as we have described previously [18].

### Targeted deep sequencing and data analysis

Ion AmpliSeq™ Designer software (Thermo Fisher Scientific Inc.) was used to design primers that covered the exons of 53 lung cancer-related genes (Supplementary Table S4) reported by The Cancer Genome Atlas Project [19, 20]. Targeted deep sequencing was performed as previously described [21]. Briefly, sequencing libraries were prepared using the Ion AmpliSeq™ Library Kit 2.0 (Thermo Fisher Scientific Inc.). Emulsion PCR was performed using the Ion OneTouch system and Ion PI™ Template OT2 200 Kit v3 (Thermo Fisher Scientific Inc.). Template-positive Ion Sphere™ Particles were enriched using the Ion OneTouch system and loaded onto an Ion PI Chip v2. Massively parallel sequencing was performed using an Ion Proton™ instrument and the Ion PI™ Sequencing 200 Kit v3 (Thermo Fisher Scientific Inc.). Single nucleotide variants, insertions, and deletions were annotated using the Ion Reporter Server System (Thermo Fisher Scientific Inc.). Lymphocytes from peripheral blood DNA were used as controls to detect variants in tumors [22]. Mutations with a variant allele fraction of ≥1.0% were considered confidence-based somatic mutations; these were also examined in bronchial wash fluid, sputum, and plasma specimens. Sequencing data were visualized using Integrative Genomics Viewer [23].

### Statistical analyses

Continuous variables are presented as the means and standard deviations. Categorical variables were compared between the groups using the Chi-square test. A one-way analysis of variance and Tukey-Kramer multiple comparisons test were used to assess statistical significance. Multivariate analyses and calculations of odds ratios and 95.0% CIs were performed using JMP software (SAS Institute, Cary, NC, USA). A two-tailed *P* < 0.05 was considered statistically significant.

## SUPPLEMENTARY MATERIALS TABLES



## Abbreviations

CI: confidence interval
CT: computed tomography
ctDNA: circulating tumor DNA
EGFR: epidermal growth factor receptor
FFPE: formalin-fixed paraffin-embedded
