# Supplementary material for: Detection of tumor-derived DNA dispersed in the airway improves the diagnostic accuracy of bronchoscopy for lung cancer
Source: Oncotarget. 2017 May 24;8(45):79404–13. doi: 10.18632/oncotarget.18159 (PMC5668052; doi:10.18632/oncotarget.18159)
Supplement: Supplementary file 1 [file oncotarget-08-79404-s001.docx]

**Supplementary Table 1.** Characteristics of patients with and without a correct diagnosis

|  | patients with correct diagnosis | patients without correct diagnosis | *p* value |
| --- | --- | --- | --- |
| Number of patients Age  Male/Female Size  く 2 cm 2 - 3 cm  > 3 cm  Histology  adenocarcinoma squamous cell carcinoma others  Location  Central Middle Peripheral  Pathological stage  I II III IV | 116 | 75 |  |
|  | 70.3 ± 10.4a | 70.6 ± 6.4a | 0.89b |
|  | 68/46 | 47/28 | 0.68c |
|  |  |  | <0.05c |
|  | 12 | 29 |  |
|  | 39 | 32 |  |
|  | 64 | 15 |  |
|  |  |  | 0.81c |
|  | 90 | 61 |  |
|  | 21 | 11 |  |
|  | 5 | 3 |  |
|  |  |  | <0.05c |
|  | 16 | 4 |  |
|  | 31 | 11 |  |
|  | 69 | 60 |  |
|  |  |  | 0.71c |
|  | 70 | 45 |  |
|  | 10 | 9 |  |
|  | 32 | 20 |  |
|  | 4 | 1 |  |

^a^Mean±SD, ^b^unpaired Student's *t* test, ^c^χ^2^ test.

**Supplementary Table 2.** Characteristics of patients in the genomic study.

|  | **Age** | **Gender** | **Smoking** | **Operative procedure** | **Size(mm) (mm)** | **pTNM** | **p-stage** | **Hisopathology** |
| --- | --- | --- | --- | --- | --- | --- | --- | --- |
| 1 | 64 | M | former | right upper lobectomy, ND2a-ii | 3O | 1a-O-O | IA | invasive Ad |
| 2 | 5O | M | former | right lower lobectomy, ND2a-ii | 18 | 1a-O-O | IA | invasive Ad |
| 3 | 71 | M | current | right upper lobectomy, ND2a-ii | 3O | 1a-O-O | IA | invasive Ad |
| 4 | 69 | M | former | left lower lobectomy, ND2a-ii | 2O | 1a-1-O | IIA | invasive Ad |
| 5 | 65 | M | current | left upper lobectomy, ND2a-ii | 16 | 1a-O-O | IA | adenocarcinoma in situ |
| 6 | 75 | M | former | right upper lobectomy, ND2a-ii | 28 | 1a-O-O | IA | squamous cell carcinoma |
| 7 | 68 | F | never | right lower lobectomy, ND2a-ii | 1O | 1a-O-O | IA | adenocarcinoma in situ |
| 8 | 6O | F | former | right lower lobectomy, ND2a-ii | 22 | 1a-O-O | IA | invasive Ad |
| 9 | 63 | M | former | left lower lobectomy, ND2a-ii | 25 | 1a-O-O | IA | invasive Ad |
| 1O | 75 | M | former | right upper lobectomy, ND2a-ii | 3O | 2a-O-O | I8 | invasive Ad |
| 11 | 74 | M | former | right upper lobectomy, ND2a-ii | 15 | 1a-O-O | IA | squamous cell carcinoma |
| 12 | 72 | M | current | left upper lobectomy, ND2a-ii | 25 | 1b-O-O | IA | invasive Ad |
| 13 | 84 | M | former | left upper lobectomy, ND2a-ii | 3O | 1b-O-O | IA | squamous cell carcinoma |
| 14 | 83 | M | current | right lower lobectomy, ND2a-ii | 3O | 2a-O-O | I8 | small cell carcinoma |
| 15 | 72 | M | former | right lower lobectomy, ND2a-ii | 15 | 1a-O-O | IA | invasive Ad |
| 16 | 78 | F | never | left upper lobectomy, ND2a-ii | 22 | 1b-O-O | IA | invasive Ad |
| 17 | 63 | M | former | middle lobectomy, ND2a-ii | 15 | 1a-O-O | IA | invasive Ad |
| 18 | 75 | F | never | right lower lobectomy, ND2a-ii | 1O | 1a-O-O | IA | invasive Ad |

M: male, F: female, ND: node dissection, Ad: adenocarcinoma.

**Supplementary Table 3.**  Circulating or dispersed tumor DNA.

| Case | mutated gene (amino acid) | Tumor | Sputum | Plasma | Br-W sup. | Br-W ppt. |
| --- | --- | --- | --- | --- | --- | --- |
| 1 | RBM10(p.Phe243fs) | 73% |  |  | 4% | 3% |
|  | EGFR(p.Leu858Arg) | 35% |  |  | 1.0% | 2% |
|  | PIK3CA(p.Cys420Arg) | 25% |  |  |  |  |
|  | ARID1B(p.Gln763Ter) | 11% |  |  |  |  |
|  | NOTCH1(p.Cys166Tyr) | 4% |  |  |  |  |
|  | EGFR(p.Thr790Met) | 1% |  |  |  | 0.3% |
| 2 | EGFR(p.Leu858Arg) | 68% |  |  | 4% |  |
|  | TP53(p.Gly245Ser) | 60% |  |  | 2% |  |
| 3 | TP53(p.Gln317Ter) | 82% | 7% | 2% | 21% |  |
|  | COBL(p.Pro828Thr) | 15% | 4% | 2% | 5% |  |
| 4 | KEAP1(p.Tyr443Cys) | 54% |  |  |  |  |
|  | KRAS(p.Gly12Asp) | 53% |  |  |  |  |
| 5 | TP53(p.Cys277Phe) | 40% |  |  |  |  |
|  | EGFR(p.Leu858Arg) | 34% |  |  |  |  |
| 6 | TP63(p.Ser38Ala) | 17% |  |  |  |  |
| 7 | EGFR(p.Leu858Arg) | 55% |  |  |  |  |
| 8 | TP53(p.Trp146Ter) | 7% |  |  |  |  |
|  | EGFR(p.Gly719Ala) | 5% |  |  | 0.7% |  |
| 9 | RBM10(p.Met104fs) | 55% |  |  |  | 1.0% |
|  | EGFR(p.Leu858Arg) | 31% |  |  | 0.3% | 0.3% |
| 10 | RBM10 (p.Cys826Ser) | 56% |  |  | 2% | 2% |
|  | RASA1 (p.Asn461Asp) | 38% |  |  |  |  |
|  | ARID1B (p.Met931Val) | 34% |  |  |  |  |
| 11 | KEAP1(p.His247Arg) | 75% |  |  |  | 1.1% |
|  | TP53(p.Asn239Ser) | 66% |  |  | 2% | 1% |
| 12 | RBM10(p.Gly508fs) | 57% |  |  |  |  |
|  | CDKN2A(p.Trp110Ter) | 30% |  |  |  |  |
| 13 | NFE2L2(p.Asp29Gly) | 69% |  |  | 10% |  |
|  | TP53(p.Arg273Leu) | 67% |  |  | 13% |  |
|  | EPHA7(p.Val561Phe) | 22% |  |  | 3% |  |
| 14 | TP53(p.Cys242Phe) | 81% |  |  | 2% | 2% |
|  | RB1(p.Phe839fs) | 69% |  |  |  |  |
|  | NOTCH2(p.Ser1407Leu) | 51% |  |  | 2% | 1.4% |
|  | ATM(Splise site) | 45% |  |  | 3% | 4% |
|  | COBL(p.Leu857Val) | 44% |  |  | 2% | 1.1% |
|  | RB1(p.His686Arg) | 4% |  |  | 0.5% |  |
| 15 | KRAS(p.Gly12Asp) | 43% |  |  |  |  |
|  | TP53(Splise site) | 23% |  |  |  |  |
| 16 | TSC2(p.Glu1490Gln) | 36% |  |  |  |  |
|  | EGFR(p.Ala750Pro) | 34% |  |  |  |  |
|  | NFE2L2(p.Glu563Leu) | 31% |  |  |  |  |
| 17 | PTEN(p.Ile32del) | 15% |  |  | 1.1% |  |
|  | CDKN2A(p.Leu32Pro) | 12% |  |  | 0.8% |  |
|  | KRAS(p.Gly12Asp) | 9% |  |  | 0.6% |  |
| 18 | TP53(p.Arg282Gly) | 21% |  |  |  |  |

The data in the list indicates an allele fraction of the detected mutant gene.

Br-W: bronchial wash, sup: supernatant, ppt: precipitant.

**Supplementary Table 4.** The genes targeted in the cancer panel.

| **No** | **Gene symbol** | **Chromosome** | **Number of Amplicons** | **Total Bases** | **Covered Bases** | **Overall Coverage** |
| --- | --- | --- | --- | --- | --- | --- |
| 1 | AKT1 | chr14 | 26 | 1573 | 1497 | 95% |
| 2 | AKT2 | chr19 | 27 | 1576 | 1543 | 98% |
| 3 | AKT3 | chr1 | 30 | 1624 | 1624 | 100% |
| 4 | ARID1A | chr1 | 76 | 7058 | 6023 | 85% |
| 5 | ARID1B | chr6 | 75 | 6950 | 5965 | 86% |
| 6 | ARID2 | chr12 | 71 | 5718 | 5643 | 99% |
| 7 | ASCL4 | chr12 | 5 | 532 | 382 | 72% |
| 8 | ATM | chr11 | 147 | 9791 | 9439 | 96% |
| 9 | BRAF | chr7 | 37 | 2481 | 2224 | 90% |
| 10 | CDKN2A | chr9 | 9 | 962 | 612 | 64% |
| 11 | COBL | chr7 | 48 | 4151 | 3977 | 96% |
| 12 | CREBBP | chr16 | 96 | 7639 | 7071 | 93% |
| 13 | CTNNB1 | chr3 | 32 | 2486 | 2486 | 100% |
| 14 | CUL3 | chr2 | 42 | 2561 | 2495 | 97% |
| 15 | EGFR | chr7 | 60 | 4189 | 4135 | 99% |
| 16 | EP300 | chr22 | 90 | 7555 | 7182 | 95% |
| 17 | EPHA7 | chr6 | 44 | 3175 | 3154 | 99% |
| 18 | ERBB2 | chr17 | 57 | 4080 | 3808 | 93% |
| 19 | ERBB3 | chr12 | 59 | 4440 | 4374 | 99% |
| 20 | FGFR1 | chr8 | 41 | 2825 | 2816 | 100% |
| 21 | FGFR2 | chr10 | 43 | 2910 | 2842 | 98% |
| 22 | FGFR3 | chr4 | 34 | 2752 | 2215 | 81% |
| 23 | FOXP2 | chr7 | 36 | 2487 | 2469 | 99% |
| 24 | HRAS | chr11 | 11 | 683 | 683 | 100% |
| 25 | KEAP1 | chr19 | 24 | 1925 | 1845 | 96% |
| 26 | KMT2D | chr12 | 192 | 17154 | 15854 | 92% |
| 27 | KRAS | chr12 | 10 | 737 | 681 | 92% |
| 28 | MAP2K1 | chr15 | 18 | 1292 | 1239 | 96% |
| 29 | MET | chr7 | 59 | 4427 | 4396 | 99% |
| 30 | MGA | chr15 | 110 | 9428 | 9345 | 99% |
| 31 | MLL | chr11 | 144 | 12279 | 11875 | 97% |
| 32 | NF1 | chr17 | 136 | 9161 | 9023 | 99% |
| 33 | NFE2L2 | chr2 | 23 | 1868 | 1826 | 98% |
| 34 | NOTCH1 | chr9 | 99 | 8008 | 7078 | 88% |
| 35 | NOTCH2 | chr1 | 101 | 7809 | 7539 | 97% |
| 36 | NRAS | chr1 | 9 | 610 | 610 | 100% |
| 37 | PIK3CA | chr3 | 50 | 3407 | 3282 | 96% |
| 38 | PTEN | chr10 | 18 | 1302 | 1223 | 94% |
| 39 | RASA1 | chr5 | 55 | 3412 | 3216 | 94% |
| 40 | RB1 | chr13 | 55 | 3057 | 2902 | 95% |
| 41 | RBM10 | chrX | 48 | 3228 | 3079 | 95% |
| 42 | RIT1 | chr1 | 13 | 771 | 771 | 100% |
| 43 | SETD2 | chr3 | 91 | 7905 | 7663 | 97% |
| 44 | SLIT2 | chr4 | 76 | 4972 | 4854 | 98% |
| 45 | SMAD4 | chr18 | 24 | 1769 | 1715 | 97% |
| 46 | SMARCA4 | chr19 | 74 | 5399 | 5055 | 94% |
| 47 | SOX2 | chr3 | 9 | 964 | 883 | 92% |
| 48 | STK11 | chr19 | 23 | 1392 | 1343 | 97% |
| 49 | TP53 | chr17 | 22 | 1383 | 1351 | 98% |
| 50 | TP63 | chr3 | 34 | 2360 | 2227 | 94% |
| 51 | TSC1 | chr9 | 49 | 3705 | 3603 | 97% |
| 52 | TSC2 | chr16 | 92 | 5834 | 5677 | 97% |
| 53 | U2AF1 | chr21 | 15 | 880 | 870 | 99% |
